# STFDSGCN: Spatio-Temporal Fusion Graph Neural Network Based on Dynamic Sparse Graph Convolution GRU for Traffic Flow Forecast

**DOI:** 10.3390/s25113446

**Published:** 2025-05-30

**Authors:** Jiahao Chang, Jiali Yin, Yanrong Hao, Chengxin Gao

**Affiliations:** 1 College of Software, Taiyuan University of Technology, Taiyuan 030024, China; 2 School of Computer Science and Technology, Taiyuan University of Science and Technology, Taiyuan 030024, China

**Keywords:** traffic flow forecasting, graph neural networks (GNN), gated recurrent units (GRUs), dynamic sparse graph convolution, spatio-temporal attention

## Abstract

The characteristics of multivariate heterogeneity in traffic flow forecasting exhibit significant variation, heavily influenced by spatio-temporal dynamics and unforeseen events. To address this challenge, we propose a spatio-temporal fusion graph neural network based on dynamic sparse graph convolution GRU for traffic flow forecast (STFDSGCN), which incorporates a spatio-temporal attention fusion scheme with a gating mechanism. The dynamic sparse graph convolution gated recurrent unit (DSGCN-GRU) in this model is a novel component that integrates adaptive dynamic sparse graph convolution into the gated recurrent network to simulate the diffusion of information within a dynamic spatial structure. This approach effectively captures the heterogeneous and local features of spatial data, further reflecting the irregularities and dynamic variability inherent in spatial information. By leveraging spatio-temporal attention through the gating mechanism, the model enhances its understanding of both local and global spatio-temporal characteristics. This enables a unified representation of multi-scale and long-range spatio-temporal patterns and strengthens the model’s ability to respond to long-term traffic flow forecasting and traffic emergencies. Extensive experiments on two real-world datasets demonstrate that, compared to advanced methods that lack sufficient multivariate heterogeneous feature extraction and do not account for traffic emergencies, the STFDSGCN model improves the average absolute error (MAE), root mean square error (RMSE), and average absolute percentage error (MAPE) by 4.01%, 1.33%, and 1.03%, respectively, achieving superior performance.

## 1. Introduction

In recent years, with the rapid urbanization and a significant increase in the number of motor vehicles, traffic congestion has become a major challenge, limiting urban development and the improvement of residents’ quality of life. Intelligent transportation systems (ITSs), a key technology [1] for addressing this issue, place traffic flow prediction at their core, aiming to achieve both accuracy and efficiency. Accurate traffic flow prediction can help alleviate road congestion, optimize urban traffic network management, and enhance overall traffic efficiency [2]. The primary challenge in traffic flow prediction, however, lies in effectively integrating time-series features with the complex spatial structure of road networks. Over the past few decades, traffic flow prediction has become a pivotal research focus within the realm of intelligent transportation systems, garnering extensive scholarly attention [3].

Traffic flow forecasting plays a vital role in enabling intelligent decision-making within modern transportation systems. As urban traffic becomes increasingly dynamic and complex, accurate forecasting provides the foundation for proactive traffic management, including congestion mitigation, adaptive signal control, and route planning. Moreover, with the integration of smart city infrastructure and real-time sensing technologies, the demand for reliable and fine-grained traffic prediction models has grown rapidly. Traffic forecasting not only supports the operational efficiency of transportation agencies but also enhances road safety and environmental sustainability by reducing travel delays, fuel consumption, and emissions. Therefore, developing robust and generalizable models for traffic flow prediction is of both theoretical and practical importance, especially under conditions of uncertainty and sudden disruptions.

Traffic flow prediction seeks to forecast future traffic conditions based on historical traffic data. Early efforts in traffic flow prediction mainly relied on traditional time-series analysis models, such as the Autoregressive Integrated Moving Average (ARIMA) [4] model and the Vector Autoregression (VAR) [5] model. However, these models have limitations in handling nonlinear relationships and leveraging the spatial correlations of urban traffic networks, making them less effective for complex traffic flow predictions. To address the challenges posed by nonlinearity, researchers have turned to various machine learning methods, including Bayesian Networks [6], support vector regression (SVR) [7], and K-Nearest Neighbors (KNNs) [8]. While these approaches have been instrumental in uncovering the nonlinear characteristics of traffic flow, they often require labor-intensive manual feature selection and construction, which can be both costly and susceptible to subjective bias.

With the rapid advancement of deep learning technologies, traffic flow prediction research has increasingly shifted towards neural network architectures. Significant advancements have been made in traffic flow forecasting, owing to its unparalleled capacity for automatic feature extraction from raw data [9]. Recurrent neural networks (RNNs) [10] and their variants, such as Long Short-Term Memory (LSTM) [11,12] and gated recurrent units (GRUs) [13], have demonstrated significant advantages in capturing the temporal dynamics of traffic flow. Notably, Ma et al. [14] pioneered the use of LSTM networks for traffic speed prediction, while Tian et al. [15] employed stacked bidirectional and unidirectional LSTMs to model bidirectional temporal dependencies. However, RNN-based methods primarily focus on the temporal variability of the data, often neglecting the spatial correlations inherent in traffic systems. To address this limitation, Convolutional Neural Networks (CNNs) were introduced to capture spatial dependencies in traffic data structured on Euclidean grids [16]. For example, Zhang et al. [17] proposed ST-ResNet, a CNN-based model for urban pedestrian flow prediction. Additionally, CNN-based architectures such as temporal convolutional networks (TCNs) [18,19] and Graph WaveNet [20] have been utilized to overcome the sequential limitations of RNNs and enhance the modeling of temporal dependencies. However, these models still fall short in capturing the spatial irregularities of real-world traffic networks, which are fundamentally non-Euclidean in nature [21,22].

To better model the complex and irregular topologies of traffic systems, graph neural networks (GNNs) have gained increasing attention for their ability to process non-Euclidean structured data and capture intricate spatial dependencies. GNN-based approaches have become a core technology in traffic forecasting research [23,24], as they naturally align with the spatial structure of road networks. Among them, STGCN [25] marked a significant milestone by pioneering the use of graph convolution to model spatio-temporal dependencies. Subsequent models built upon this foundation: DCRNN [26] combined RNNs with diffusion graph convolution; GMAN [27] introduced multi-attention mechanisms.

More recent advancements have focused on refining the representation of dynamic spatio-temporal features [28]. ASTGCN [29] incorporated spatial and temporal attention into the GCN framework to better capture subtle variations, while STSGCN [30] applied a sliding window strategy to learn local spatio-temporal graphs. STFGNN [31] introduced a parallel fusion of spatio-temporal features, and DSTAGNN [32] replaced static graphs with dynamic spatio-temporal-aware graphs, addressing limitations of fixed structures. Further studies [33,34,35] proposed innovative dynamic graph construction methods to adapt to the evolving nature of traffic networks and improve predictive accuracy.

Despite these advancements, many existing methods still rely on predefined or static graph structures, which restrict their ability to fully reflect the dynamic changes and multivariate heterogeneity of real-world traffic systems. This becomes especially critical when modeling the interactions among diverse features, managing complex spatio-temporal dependencies, and responding to sudden and unpredictable events. For instance, while STSGCN [30] focuses on evolving spatial patterns, it does not explicitly address temporal dynamics. AGCRN [36], which integrates adaptive graph construction with recurrent neural networks, improves representation capability but lacks comprehensive global information modeling.

Traffic flow is a typically complex system that is influenced not only by temporal factors (such as regular fluctuations during rush hours or holiday effects) and spatial factors (such as geographic location and road network structure) but also by various other elements such as traffic accidents, temporary road closures, and large-scale events. These factors contribute to the “heterogeneous characteristics” in traffic flow prediction, and their interactions and feedback mechanisms form a dynamically changing network. However, in such a complex and dynamic traffic environment, previous methods [37,38,39,40] have not addressed the impact of sudden traffic events on traffic flow prediction. As a result, these models struggle to effectively handle unexpected traffic incidents.

To address the limitations in extracting spatiotemporal heterogeneous features and to effectively tackle the challenges arising from dynamic traffic conditions and sudden incidents, this paper spatio-temporal fusion graph neural network based on dynamic sparse graph convolution GRU for traffic flow forecast (STFDSGCN). We enhance the spatiotemporal features of the input data through an effective data preprocessing layer. Furthermore, we introduce a novel structure, the dynamic sparse graph convolutional gated recurrent network (DSGCN-GRU), which is designed to simultaneously capture local dynamic spatiotemporal features. To further improve model performance, we incorporate a dual attention mechanism, combining gated temporal attention and spatial attention, to address the DSGCN-GRU’s shortcomings in handling anomalies and long-range dependencies. In summary, the key contributions of STFDSGCN are:

- The STFDSGCN model, which integrates a gated recurrent network architecture with a combined attention mechanism, enabling effective extraction of spatiotemporal features for accurate traffic flow prediction.
- The introduction of a novel dynamic sparse graph convolutional gated recurrent network (DSGCN-GRU), a unique module that embeds adaptive sparse graph convolution and graph attention blocks into gated recurrent units, allowing for the capture of local temporal dependencies and spatially heterogeneous characteristics.
- The incorporation of dual attention mechanisms—gated temporal attention and spatial attention—enhancing the model’s capability to understand complex spatiotemporal patterns. This fusion improves the model’s ability to capture long-range dependencies, thus increasing the accuracy of long-term traffic flow predictions and strengthening its responsiveness to sudden changes in traffic conditions.

## 2. Methods

### 2.1. Data Structure Definition

Definition (traffic graph): the road traffic network, monitored by a variety of sensors, is represented as a graph G=(V,E,A), where $V=(v_{1},v_{2},\ldots ,v_{N})$ denotes the set of nodes and E represents the set of edges. The adjacency matrix $A\in R^{N\times N}$ captures the connectivity between the nodes. Additionally, the matrix $D\in R^{N\times N}$ is a weighted adjacency matrix that quantifies the specific weight relationships among the nodes. The edges in this graph signify the connections between the nodes, and the weights assigned to these edges reflect the distances separating the nodes.

Definition (traffic flow): traffic flow is defined as the quantity of vehicles or pedestrians passing through designated locations, such as roads or intersections, within a specific time frame. It provides insights into traffic conditions and is instrumental in determining congestion levels. In the framework of this study, the traffic flow at time t in a road network with N nodes is represented by $X_{t}\in R^{N\times C}$, where C denotes the number of traffic flow features. Moreover, the signals across all nodes during a time interval T are denoted as $X=(X_{1},X_{2},\ldots ,X_{N})\in R^{T\times N\times C}$.

Definition (traffic flow prediction): the task of traffic flow prediction involves forecasting future traffic conditions based on historical traffic data. The data for the previous T time steps is represented as $P=(X_{\theta -T+1},X_{\theta -T+2},\ldots ,X_{\theta })\in R^{N\times C\times T}$, while the forecasted data for the subsequent $T^{\prime }$ time steps is expressed as $Q=(X_{\theta +1},X_{\theta +2},\ldots ,X_{\theta +T^{\prime }})\in R^{N\times C\times T^{\prime }}$. Mathematically, the prediction task can be seen as mapping the historical observations P to the predicted future state Q, in the form:

(1) $$Q=f\left(P;G\right)$$

In this context, f represents the mapping function that learns to transform the historical sequence into the predicted sequence. The primary focus of this paper is on the development and optimization of this mapping function to enhance both the accuracy and practical applicability of the predictions.

### 2.2. Overall Structure of the Model

**Figure 1:**
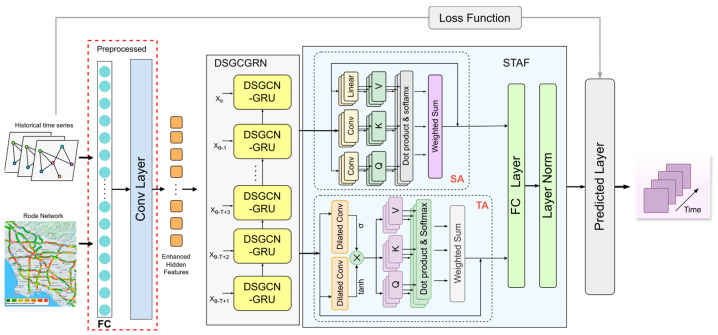
The overall architecture of STFDSGCN.

The STFDSGCN model’s overall architecture is illustrated in Figure 1. It is primarily composed of two key components: the dynamic sparse graph convolutional gated recurrent network (DSGCGRN) and the spatio-temporal attention fusion layer (STAF). In Figure 1, the DSGCGRN consists of multiple DSGCN-GRU layers, where each layer processes the output of the previous layer to generate a new hidden state. The STAF, as depicted in Figure 1, includes a Temporal Multi-Head Attention Block (TA) with dilated convolution gating and a Spatial Multi-Head Attention Block (SA). Initially, traffic flow data $P=(X_{\theta -T+1},X_{\theta -T+2},\ldots ,X_{\theta })\in R^{N\times C\times T}$ is input into the model. After passing through fully connected layers and convolutional layers [41], the model produces an enhanced spatio-temporal feature representation, which is computed as follows:

(2) $$X_{FC}=W_{2}\left(ReLU\left(W_{1}\left(P\right)\right)\right)⊙P$$

(3) $$X_{t}^{H}=Sigmoid\left(Conv\left(ReLU\left(Conv\left(X_{FC}\right)\right)\right)\right)⊙X_{FC}$$

The matrices $W_{1}$ and $W_{2}$ represent the weight matrices for the first and second linear layers of the fully connected layer, respectively. The activation functions used are *ReLU* and *Sigmoid*, while ⊙ denotes element-wise multiplication. The matrix $X_{t}^{H}=(X_{\theta -T+1}^{H},X_{\theta -T+2}^{H},\ldots ,X_{\theta }^{H})\in R^{N\times C\times T}$ represents the enhanced hidden feature representation. This is then input $X_{t}^{H}\in R^{N\times C\times T}$ into the dynamic sparse graph convolutional recurrent network to capture local spatio-temporal correlations. The DSGCGRN module combines the gated recurrent unit (GRU) with dynamic sparse graph convolution (DSGCN) to effectively process spatio-temporal data, taking into account its irregularities and dynamic variations. The output from the DSGCN-GRU is subsequently passed into the attention fusion layer, which employs spatial attention (SA) and temporal attention (TA) to handle different types of information. This enhances the model’s capacity to understand both local and global spatio-temporal features, improving its ability to predict long-term traffic flow and respond to sudden traffic events. Finally, the output of the attention fusion layer is fed into the prediction layer, where it interacts with the loss function during training to generate predictions for future traffic data $Q\in R^{N\times C\times T}$.

### 2.3. Dynamic Sparse Graph Convolutional Recurrent Network (DSGCGRN)

**Figure 2:**
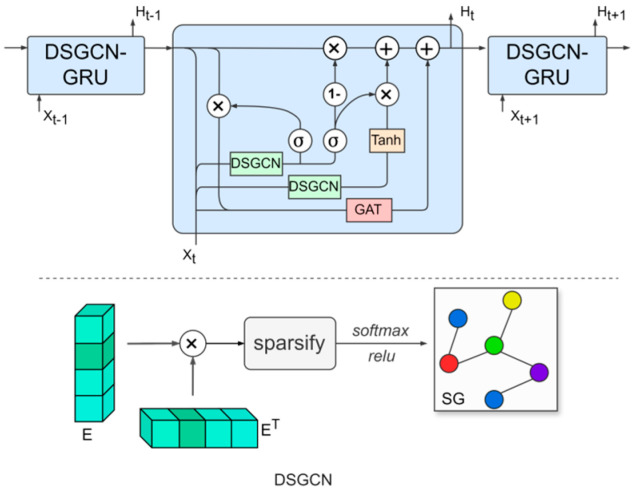
Gated Recurrent Network with Dynamic Sparse Graph Convolution.

In real-world traffic networks, the mutual influence between traffic flows is not constant but varies over time, leading to significant correlations between roads due to dynamic traffic flow fluctuations. To capture and understand the spatial correlations in traffic data, we employ graph convolutional networks (GCNs) to model the information transmission process within dynamic spatial structures [42], thereby obtaining feature representations of nodes. However, traditional GCNs are limited in their ability to handle static graph structures, which restricts their capacity to capture dynamic spatial correlations. In contrast, dynamic GCNs can adaptively generate adjacency matrices that reflect these time-varying relationships, allowing for a more accurate representation of spatial correlations in traffic flow across different time periods. To address this challenge, we propose the dynamic sparse graph convolutional gated recurrent network (DSGCGRN), as illustrated in Figure 2.

The DSGCGRN consists of multiple layers of DSGCN-GRU structures, with each GRU unit incorporating two novel dynamic sparse graph convolution blocks and one graph attention block. By combining the strengths of dynamic sparse graph convolution (DSGCN) and gated recurrent units (GRUs), the model is able to capture both spatial and temporal correlations. The DSGCN module dynamically updates the adjacency matrix based on the evolving traffic data, thereby capturing the shifting spatial correlations in the traffic network over time. The sparse graph learning approach encourages the model to identify key, explicit connections, effectively limiting the spatial receptive field to preserve the distinctive characteristics of individual nodes [43]. Meanwhile, the GRU component handles the processing and storage of time-series information.

First, we generate adaptive node embeddings to calculate the adjacency matrix $A\in R^{N\times N}$. Next, we apply a sparsification technique to A, keeping only the top highest similarity connections between each node and its neighboring nodes. The resulting matrix is then normalized using the softmax function to obtain the adaptive sparse adjacency matrix $S_{A}\in R^{N\times N}$. The formula for this process is defined as:

(4) $$A=ReLU\left(EE^{T}\right)$$

(5) $$S_{A}=softmax\left(ReLU\left(sparsify\left(A;k\right)\right)\right)$$

Here, $E\in R^{N\times d}$ is the adaptive node embedding matrix, where d denotes the embedding dimension of the nodes. The function sparsify(*) denotes the sparsification operation, and k is the sparsity rate.

To enable our method to learn higher-order neighborhood information in graph data, we utilize Chebyshev polynomials [44] to approximate the graph convolution kernel. This technique allows us to efficiently model the intricate relationships between nodes and their distant neighbors, as well as the broader global structure, without significantly increasing computational complexity. Therefore, the dynamic graph convolutional network can be defined as:

(6) $$L=\left(I+S_{A}\right)X^{H}EW+Eb$$

Here, I represents the identity matrix, $X^{H}\in R^{N\times C}$ be the input graph signal, and $L\in R^{N\times D}$ represent the output signal after the graph convolution operation. The learnable parameters of the model are $W\in R^{C\times D}$ and $b\in R^{d\times D}$.

In order to consider both the spatial topology and the interactions between nodes while also capturing temporal dependencies, we combine gated recurrent units (GRUs) with dynamic sparse graph generation. Specifically, we integrate dynamic graph convolution into the GRU with graph attention (GAT) gating. The overall structure is shown in Figure 2. Given the previous hidden state $H_{t-1}$ and the input data at time t, $X_{t}\in R^{N\times D}$, the gated recurrent unit can be expressed as follows:

(7) $$u_{t}=\sigma \left(\left[X_{t},H_{t-1}\right]{⋆}_{G}W_{u}+b_{u}\right)$$

(8) $$u_{t}=\sigma \left(\left[X_{t},H_{t-1}\right]{⋆}_{G}W_{u}+b_{r}\right)$$

(9) $$c_{t}=tanh\left(\left[X_{t},(r_{t}⊙H_{t-1}\right)\right]{⋆}_{G}W_{c}+b_{c})$$

(10) $$G_{att}=\left(M⊙A\right)X^{H}W$$

(11) $$H_{t}=u_{t}⊙H_{t-1}+\left(1-u_{t}\right)⊙c_{t}+G_{att}$$

Here, ${⋆}_{G}$ indicates the graph convolution operation, σ represents the sigmoid activation function, and $W_{u}$, $b_{u}$, $W_{r}$, $b_{r}$, $W_{c}$, and $b_{c}$ are the learnable weights of the recurrent network layer. The output at time t is denoted as $H_{t}$. A is the static matrix in the GAT (graph attention network) computed based on distance, M is the dynamic attention coefficient matrix, and W represents the learnable weight parameters.

### 2.4. Spatio-Temporal Attention Fusion Layer

Although the DSGCN-GRU approach, which relies on GRU, is capable of effectively capturing local temporal patterns in traffic sequence data through its internal hidden states and memory mechanisms, its forget gate and limitations in the direction of information flow hinder its ability to detect long-range spatio-temporal dependencies. Therefore, we propose a spatio-temporal attention fusion (STAF) structure to further process the output $H_{t}\in R^{N\times T\times D}$ of the DSGCN-GRU network, aiming to capture long-range, multi-scale spatio-temporal patterns.

**Figure 3:**
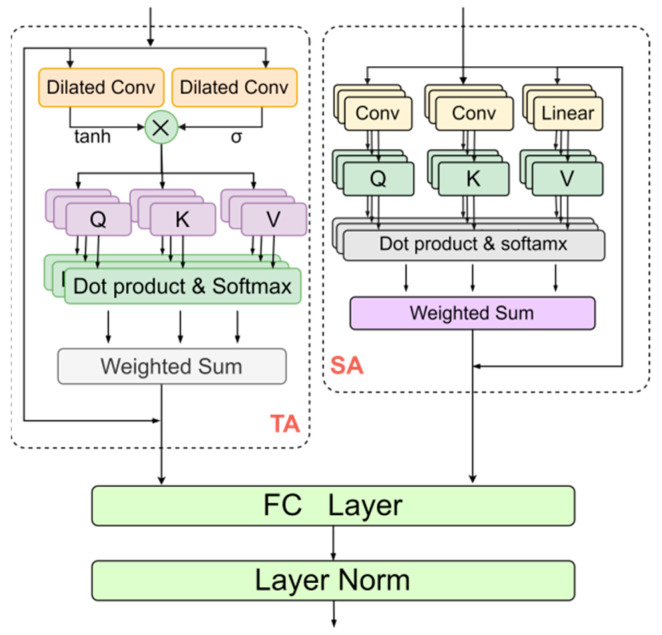
Spatio-temporal attention fusion structure.

The spatio-temporal attention fusion (STAF) structure employs a parallel design, comprising a Temporal Multi-Head Attention block (TA) with dilated convolution gating and a Spatial Multi-Head Attention block (SA), as illustrated in Figure 3. This design is intended to overcome the limitations of recurrent neural networks in processing long spatio-temporal sequences and their inability to efficiently handle sudden traffic events. Initially, the output from the DSGCN-GRU network is separately input into the TA and SA blocks to capture long-range temporal and spatial dependencies, as well as to detect local abrupt changes. The outputs of both blocks are then combined using a fully connected layer and layer normalization [45]. The following sections offer a detailed discussion of these two modules.

#### 2.4.1. Temporal Dilated Gated Multi-Head Self-Attention

In the temporal dimension, we propose an innovative integration of dilated convolutional gating with temporal attention to assess the significance of information propagation before passing it to the multi-head attention layer. The specifics of this approach are outlined in the TA block in Figure 3. The Temporal Dilated Gated Multi-Head Self-Attention block (TA) consists primarily of a dilated convolution gating mechanism combined with a temporal attention mechanism. First, we apply the dilated convolutional gating structure to dynamically regulate the model’s learning and response to spatio-temporal features, effectively capturing both local and global patterns. This design enhances flexibility, enabling the model to better adapt to sudden changes in traffic conditions. The process is defined as follows:

(12) $$filter=tanh\left(D\_conv\left(H_{t}\right)\right)$$

(13) $$gate=\sigma \left(D\_conv\left(H_{t}\right)\right)$$

(14) $$X_{tg}=filter⊙gate$$

In this context, σ denotes the sigmoid activation function and $X_{tg}$ represents the input to the subsequent temporal multi-head attention layer [46]. Next, we utilize the multi-head attention mechanism to capture dynamic temporal relationships, allowing the model to learn intricate temporal dependencies. The query Q, key K, and value V matrices are derived through linear layers to compute the attention scores. This is mathematically expressed as follows:

(15) $$Q_{T},K_{T},V_{T}=Linear\left(X_{tg}\right)$$

(16) $$Attention\left(Q_{T},K_{T},V_{T}\right)=softmax\left(\frac{Q_{T}K_{T}^{T}}{\sqrt{D}}\right)V_{T}$$

(17) $$head_{i}^{T}=Attention\left(Q_{S}W_{i}^{Q},K_{S}W_{i}^{K},V_{S}W_{i}^{V}\right)$$

Here $W_{i}^{Q},W_{i}^{K},W_{i}^{V}\in R^{d_{h}\times d_{h}}$ represents the learnable parameter matrices. The output of the Temporal Dilated Gated Self-Attention, denoted as $MSA_{T}\in R^{N\times T\times D}$ is then computed as follows:

(18) $$MSA_{T}\left(Q_{T},K_{T},V_{T}\right)=\sum_{i}^{h}head_{i}^{T}W^{T}$$

#### 2.4.2. Spatial Multi-Head Self-Attention

In the spatial dimension, unlike the TA block, we directly use the output of the Dynamic Sparse Graph Convolutional Recurrent Network to calculate the query Q, key K, and value V matrices in order to obtain the spatial attention output, as detailed in the SA block in Figure 3. Notably, we apply convolution operations to obtain Q and K, which are defined as follows:

(19) $$Q_{S},K_{S}=conv\left(H_{t}\right)$$

(20) $$V_{S}=Linear\left(H_{t}\right)$$

This method successfully captures both local and global dependencies, facilitating the efficient integration and compression of spatial information. The attention scores, along with the final output, are calculated using the multi-head self-attention mechanism, as expressed below:

(21) $$Attention\left(Q_{S},K_{S},V_{S}\right)=softmax\left(\frac{Q_{S}K_{S}^{T}}{\sqrt{D}}\right)V_{S}$$

(22) $$head_{i}^{S}=Attention\left(Q_{S}W_{i}^{Q},K_{S}W_{i}^{K},V_{S}W_{i}^{V}\right)$$

(23) $$MSA_{S}\left(Q_{S},K_{S},V_{S}\right)=\sum_{i}^{h}heade_{i}^{S}W^{S}$$

Here $W_{i}^{Q},W_{i}^{K},W_{i}^{V}\in R^{d_{h}\times d_{h}}$ are the learnable parameter matrices. $MSA_{S}\in R^{N\times T\times D}$ is the output of spatial attention.

The final output of the STAF layer is defined as follows:

(24) $$X_{F}=LN\left(FC\left(MSA_{T}+MSA_{S}\right)\right)$$

FC(*) represents the fully connected layer operation, and LN(*) represents the layer normalization operation. $X_{F}\in R^{N\times T\times D}$ represents the output of the spatio-temporal attention fusion (STAF) layer.

### 2.5. Prediction and Loss Function

Our model can generate accurate predictions through a simple and efficient convolutional prediction layer. Specifically, we map the high-dimensional spatio-temporal features obtained from the spatio-temporal attention mechanism to a lower-dimensional space using a convolutional layer to predict future traffic sequences $Q\in R^{N\times C\times T}$. In this process, since traffic data frequently includes outliers caused by sensor malfunctions or other factors, we employ the L1 loss function to assess the deviation between predicted and actual values. The L1 loss is less affected by extreme errors, enhancing the robustness of the model. The loss function is defined as follows:

(25) $$Loss\left(y_{i},{\hat{y}}_{i}\right)=y_{i}-{\hat{y}}_{i}$$

## 3. Experiment

### 3.1. Datasets

In conducting our research experiments, we rigorously used two well-known and widely applied public datasets, PeMS04 and PeMS08, as the foundation for evaluating model performance. The data used in this study are sourced from the Performance Measurement System (PeMS) of the California Department of Transportation. This system gathers and compiles data at five-minute intervals through a network of sensors installed across the road network, capturing essential traffic parameters such as vehicle speed, traffic volume, and lane occupancy. A comprehensive statistical summary of the datasets is provided in Table 1, offering detailed insights into the data’s background and structure.

### 3.2. Baseline Models

Traditional time series forecasting methods: ARIMA [4]: This model is based on the autoregressive moving average model and adds a difference term to predict the trend of traffic flow. SVR [7]: The model uses the linear support vector regression algorithm to realize the prediction function of continuous values.

Graph neural network-based approaches: STGCN [25]: For the first time, convolution operations are used to capture temporal relationships, and GCN is used to capture spatial relationships. DCRNN [26]: The diffusion convolutional recurrent neural network follows the encoder-decoder structure and uses the combination of diffusion map convolutional network and GRU to predict traffic flow. GraphWaveNet [20]: Graph WaveNet combines graph convolution with temporal convolution to capture spatiotemporal dependencies. ST-AE [37]: An autoencoder-based traffic flow prediction method, which realizes traffic flow prediction by encoding the current traffic flow into a low-dimensional representation and directly predicting the future hidden state and then decoding and reconstructing. MSTGCN [47]: A spatiotemporal deep learning framework with data normalization, a multi-semantic graph convolutional network, and external feature fusion is proposed to solve the data imbalance and complex spatiotemporal feature modeling problems in the traffic flow prediction of highway toll stations. ASTGCN [32]: The attention mechanism was introduced into traffic flow prediction, and the spatial attention and temporal attention mechanisms were designed to model the spatial sum. Temporal dynamics to construct a convolutional network of spatiotemporal graphs. STSGCN [30]: a novel spatiotemporal synchronous graph convolutional network, which uses convolution operations to capture local spatial and temporal correlations at the same time. AGCRN [36]: The model combines a circular architecture with dynamic components to automatically learn multi-level spatiotemporal dependencies in traffic data. STFGNN [33]: A time graph that fuses time and space modules is proposed for flow prediction. STGODE [48]: The algorithm uses tensor ODE to model the spatiotemporal evolution law so as to support the construction of deeper networks and collaborative mining of spatiotemporal information. In order to enhance the modeling ability, the method introduces a semantic association matrix and adopts an optimized temporal convolution module to learn long-range time dependence. DSTAGNN [32]: A new dynamic spatiotemporal perception graph replaces the predefined static graph used by the traditional graph convolution method and considers the traffic prediction from the perspective of the dynamic graph. GSTPRN [49]: A model that integrates self-attention location map convolution, an approximate personalized propagation algorithm, and adaptive graph learning, realizing accurate spatiotemporal prediction of traffic flow through gated recirculation units. PDFormer [50]: A dynamic spatiotemporal modeling framework is proposed, which captures multi-scale spatial dependence through the spatial self-attention module and the dual-view map masking matrix and explicitly models the information propagation delay in combination with the delay-sensing feature transformation module so as to enhance the spatiotemporal representation ability of traffic flow prediction.

### 3.3. Experimental Setup and Evaluation Metrics

For this experiment, we split the datasets into training, validation, and test sets in a 7:2:1 ratio. Prior to feeding the data into the model for training, all datasets were normalized using the Min-Max normalization technique. In alignment with the baseline models, we set both the historical and prediction step sizes to 12, meaning that 12 consecutive time steps (equivalent to one hour of traffic flow) from the past are used to forecast the next 12 consecutive time steps.

The model was implemented using PyTorch 2.0, with all experiments running on an NVIDIA RTX 4090D GPU (ASUS, Shanghai, China). STFDSGCN employs the Adam optimizer with a learning rate of 0.001. The batch sizes for the PeMS04 and PeMS08 datasets are set to 4 and 64, respectively, with training conducted over 200 epochs. To mitigate overfitting, an early stopping strategy with a patience value of 20 was applied.

The model’s performance is assessed using three metrics: Mean Absolute Error (MAE), Mean Absolute Percentage Error (MAPE), and root mean square error (RMSE), defined as follows:

(26) $$\mathrm{MAE}\left(Q,\hat{Q}\right)=\frac{1}{N}\overset{N}{\underset{i=1}{\sum }}\left|Q_{i}-{\hat{Q}}_{i}\right|$$

(27) $$\mathrm{RMSE}\left(Q,\hat{Q}\right)=\sqrt{\frac{1}{N}\overset{N}{\underset{i=1}{\sum }}{\left(Q_{i}-{\hat{Q}}_{i}\right)}^{2}}$$

(28) $$\mathrm{MAPE}\left(Q,\hat{Q}\right)=\frac{1}{N}\overset{N}{\underset{i=1}{\sum }}\left|\frac{Q_{i}-{\hat{Q}}_{i}}{Q_{i}}\right|$$

In this context, N denotes the number of samples, $Q_{i}$ represents the actual value, and ${\hat{Q}}_{i}$ represents the predicted value.

### 3.4. Performance Comparison and Analysis

To demonstrate the superior performance of the STFDSGCN model, we conducted comparative performance experiments using the test sets of two real-world traffic flow datasets. Table 2 summarizes the average performance of various models in predicting the next hour for the PeMS04 and PeMS08 datasets. The goal is to showcase the advantages of our model through a comprehensive performance evaluation.

For fairness and reliability, we extensively referenced and cited official records and authoritative publications of several benchmark models. STFDSGCN significantly outperforms all baseline methods on both the PeMS04 and PeMS08 datasets in most cases. On PeMS04, STFDSGCN improves the MAE and MAPE metrics by 1.5% and 1.26%, respectively, compared to the advanced DSTAGNN method. On PeMS08, STFDSGCN enhances MAE and RMSE by 5.02% and 2.63%, respectively, outperforming the state-of-the-art ST-AE method, with a 3.8% improvement in MAPE.

Our analysis indicates that models leveraging spatio-temporal graph neural networks (GNNs) show considerable performance gains over traditional linear time series forecasting methods. This improvement stems from the ability of spatio-temporal GNNs to effectively capture and utilize the spatio-temporal correlations within the data, further validating their effectiveness in traffic flow prediction. Models such as STGCN and DCRNN, which combine both temporal and spatial features, demonstrate enhanced performance. The ASTGCN model, incorporating an attention mechanism, also excels by accurately capturing long-term temporal patterns, boosting prediction accuracy. Graph WaveNet improves the understanding of temporal dynamics by integrating diffusion graph convolution with temporal convolutional networks, offering a more effective spatio-temporal feature capture.

Although STSGCN attempts to integrate spatio-temporal information in a unified manner, its reliance on a simplified sliding window strategy for time sequence handling limits its capacity to capture temporal correlations precisely, thus reducing its overall effectiveness. The STGODE and AGCRN models break away from the constraints of fixed graph structures by utilizing the adaptive or dynamic nature of spatial correlations between nodes, enhancing predictive performance. DSTAGNN introduces a dynamic spatio-temporal graph, replacing the static graph traditionally used in graph convolutions, modeling pairwise node dependencies. While it shows strong results, STFDSGCN outperforms it.

What is striking is the prediction accuracy of PDFormer, which pays more attention to the time delay of the propagation of traffic conditions between spatial locations in the traffic system and more effectively simulates the change trend of actual traffic flow. It surpasses the model we propose in every metric. However, STFDSGCN pays more attention to the spatiotemporal heterogeneity of traffic data and the impact of traffic emergencies (i.e., traffic anomalies) on spatiotemporal changes in the modeling. Although the prediction accuracy of STFDSGCN does not surpass that of PDFormer, the model training efficiency is much better than that of PDFormer.

STFDSGCN innovatively integrates a dynamic sparse graph convolutional recurrent network to capture potential spatial correlations, allowing the model to deeply explore the heterogeneous spatial features of the road network. Additionally, it uses a gated spatio-temporal attention fusion mechanism to identify critical long-term time-series information and detect unforeseen traffic incidents. This model efficiently captures spatio-temporal correlations across the entire data range, achieving superior performance compared to baseline models.

### 3.5. Ablation Experiment

In order to assess the contribution of various components within the STFDSGCN model, we performed ablation experiments on the PeMS08 and PeMS04 datasets. For this, we created five distinct variants of the STFDSGCN model, with their specific configurations outlined in Table 3.

- STFDSGCN w/o DG_TA: removes the temporal self-attention structure with dilated gating.
- STFDSGCN w/o SA: removes the spatial self-attention structure from the model.
- STFDSGCN w/o STAF: removes the spatio-temporal attention fusion module from the model.
- STFDSGCN w/o SG: replaces the dynamic sparse graph in the graph convolution block with a dense graph.
- STFDSGCN w/o DSGCN: replaces the dynamic sparse graph convolutional network (DSGCN) in the dynamic sparse graph convolutional recurrent network with a regular GCN.

**Figure 4:**
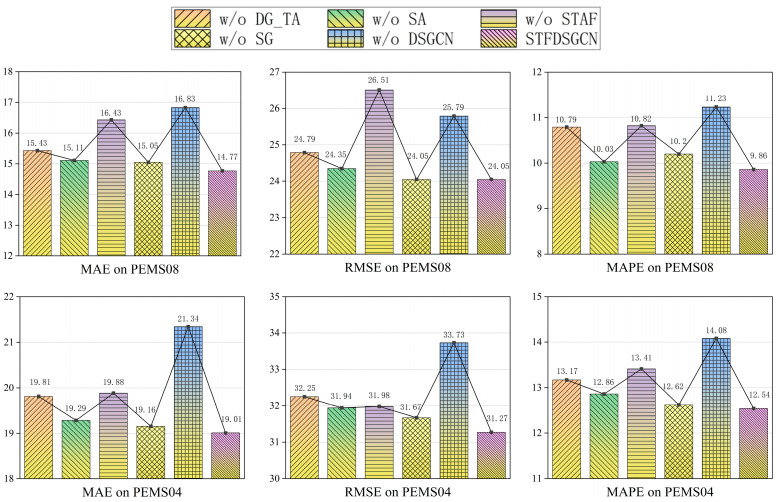
Component analysis of STFDSGCN.

Apart from the differences mentioned above, all other configurations of the variants remain consistent with STFDSGCN. Figure 4 shows the ablation results based on the PeMS08 and PeMS04 datasets. STFDSGCN demonstrates outstanding performance across all evaluation metrics. First, considering the extraction of dynamic spatial heterogeneous features, the Dynamic Sparse Graph Convolutional Recurrent Network (DSGCN) has the most significant impact on model prediction, improving MAE and MAPE by 12.24% and 12.20%, respectively. This clearly demonstrates that DSGCN, compared to the standard static GCN, is better at capturing complex and dynamic spatial correlations. The sparse graph learning approach also outperforms the dense graph as it constrains the spatial scope, preserving the uniqueness of features and thereby enhancing model performance. Second, the spatio-temporal attention fusion (STAF) mechanism contributes significantly to overall improvement, with a 10.1% increase in MAE. This mechanism balances local dependencies and global correlations in spatio-temporal features. Notably, the MAE metric is highly sensitive to outliers, and the observed significant improvement in this metric strongly confirms that this module excels in handling sudden traffic flow situations. The temporal self-attention with dilated gating and the spatial self-attention structures are both indispensable to the spatio-temporal attention fusion mechanism. Removing either would lead to a deterioration in model performance.

### 3.6. Hyperparameter Configuration Study

**Figure 5:**
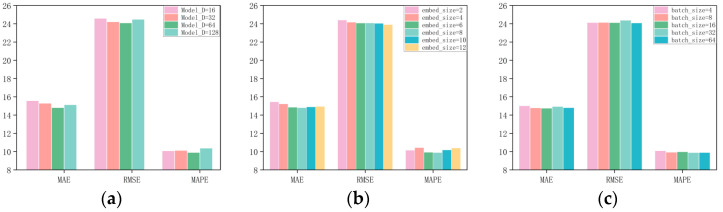
The impact of different hyperparameters on model prediction in the PeMS08 dataset. (**a**) Model dimension. (**b**) Embedding size. (**c**) Batch size.

**Figure 6:**
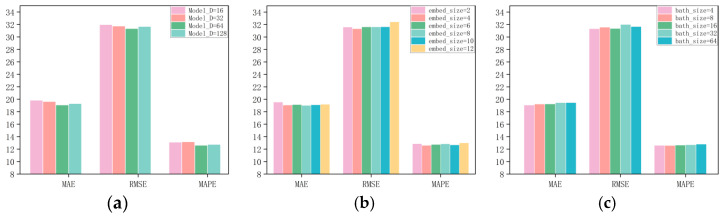
The impact of different hyperparameters on model prediction in the PeMS04 dataset. (**a**) Model dimension. (**b**) Embedding size. (**c**) Batch size.

To explore how hyperparameter choices influence model performance, we conducted a set of experiments on the PeMS04 and PeMS08 datasets with various network configurations. Our results indicated that the model’s performance was particularly sensitive to adjustments in batch size (Batch_size), model dimension (Model_D), and the dimension of node embeddings (Embedded_dim). As depicted in Figure 5a and Figure 6a, the model achieved the best predictive accuracy when the model dimension (Model_D) was set to 64. In Figure 5b and Figure 6b, it is shown that the node embedding size plays a crucial role in determining the quality of node representations in the graph. The optimal values for the node embedding dimensions were found to be 4 for PeMS04 and 8 for PeMS08. A larger node embedding dimension allows the model to accommodate more parameter information, which helps to uncover and infer more comprehensive spatial correlations. Figure 5c and Figure 6c show the influence of batch size on model performance across different datasets. The optimal `Batch_size` for PeMS04 and PeMS08 is 4 and 64, respectively. When all hyperparameters are set to their optimal values, our model produces the best performance.

### 3.7. Scalability Study

We performed a comparison of the computational costs between the STFDSGCN model and various baseline models, as shown in Table 4, using the PeMS08 dataset. While the STFDSGCN model demonstrates excellent performance, its computational cost did not increase significantly. Compared to other advanced baselines such as STGODE and DSTAGNN, STFDSGCN not only outperforms them in terms of accuracy but also significantly reduces the consumption of computational resources. While STFGNN is highly efficient and exhibits the lowest computational cost, it has some limitations in capturing long-term dependencies in time series data. PDFormer designs a more complex null attention mechanism, which leads to its complete inferiority in training efficiency compared to STFDSGCN. The main computational expense of STFDSGCN is associated with its gated spatio-temporal attention fusion structure. However, since STFDSGCN processes data in parallel within the spatio-temporal attention fusion structure, it greatly improves the model’s operational efficiency.

### 3.8. Discuss the Implications of the Study

The STFDSGCN model proposed in this study effectively solves the spatiotemporal dynamic modeling problem in traffic flow prediction through innovative dynamic sparse graph convolution and a gated spatiotemporal attention mechanism. In this study, two public datasets of California highways are used as practical application cases for traffic flow prediction, and the prediction accuracy is significantly better than that of existing methods under the influence of spatiotemporal heterogeneous characteristics and emergency response. These practical effects were significantly demonstrated in both the ablation experiment and the baseline comparison experiment. This work not only provides a new theoretical framework for spatiotemporal series prediction but also shows important engineering value and provides reliable technical support for the construction of smart cities. Future research can further optimize extreme weather adaptability and explore the deployment of edge computing to expand its application breadth in the field of intelligent transportation.

## 4. Conclusions

In this study, we proposed STFDSGCN, a novel spatio-temporal fusion graph neural network that integrates dynamic sparse graph convolution with a gated recurrent architecture and attention mechanisms, specifically designed for traffic flow forecasting. Our model demonstrates superior predictive accuracy and efficiency across two real-world highway datasets by effectively modeling heterogeneous spatio-temporal dependencies and dynamically responding to abrupt traffic conditions. Beyond technical performance, STFDSGCN offers substantial real-world value. It is particularly suited for deployment in intelligent transportation systems where both short-term accuracy and long-term adaptability are essential—such as highway congestion forecasting, real-time traffic control, and early warning systems for traffic disruptions. By requiring only fixed-location sensor data and operating with relatively low computational cost, STFDSGCN is well-positioned for integration into existing traffic management infrastructures, including edge-computing environments. While the proposed framework performs well on highway data, we acknowledge two main limitations: its current validation is restricted to highway scenarios, and it lacks direct evaluation under rare or irregular conditions such as accidents or severe weather. In future work, we plan to extend the model to more complex urban environments by incorporating mobile trajectory data such as vehicle GPS signals and vehicle-to-infrastructure communication. We also intend to draw on recent advances in urban traffic modeling, including techniques introduced in models such as PDFormer. Additionally, we will explore indirect validation strategies such as traffic anomaly detection to assess robustness under abnormal conditions and integrate external factors such as weather and public events to enhance real-time adaptability in dynamic traffic systems.

## Figures and Tables

**Table 1:** Improvements in homography transformation parameters.

Dataset	Nodes	Edges	Time Steps	Time Interval	Time Range
PeMS04	307	340	16,992	5 min	01/2018-02/2018
PeMS08	170	295	17,856	5 min	07/2016-08/2016

**Table 2:** Performance comparison of the STFDSGCN model with 15 baseline models on the PeMS04 and PeMS08 datasets.

Model	PeMS04			PeMS08		
	MAE	RMSE	MAPE (%)	MAE	RMSE	MAPE (%)
ARIMA	33.73	48.80	24.18	31.09	44.32	22.73
SVR	28.70	44.56	19.20	23.25	36.16	14.71
STGCN	21.16	34.89	13.83	17.50	27.09	11.29
DCRNN	21.22	33.44	14.17	16.82	26.36	10.92
Graph WaveNet	24.89	39.66	17.29	18.28	30.05	12.15
MSTGCN	23.96	37.21	14.33	19.00	29.15	12.38
ASTGCN	22.93	35.22	16.56	18.25	28.06	11.64
STSGCN	21.19	33.65	13.90	17.13	26.80	10.96
STFGNN	20.48	32.51	16.77	16.94	26.25	10.60
STGODE	20.84	32.82	13.77	16.81	25.97	10.62
AGCRN	19.83	32.26	12.97	15.95	25.22	10.09
ST-AE	19.68	31.28	15.14	15.55	24.70	10.25
DSTAGNN	19.30	31.46	12.70	15.67	24.77	9.94
GSTPRN	19.45	31.91	12.96	15.68	24.96	10.09
PDFormer	18.64	30.06	12.21	13.583	23.505	9.046
STFDSGCN (Ours)	19.01	31.27	12.54	14.77	24.05	9.86

**Table 3:** Ablation experiment setup. ✓ indicates that the corresponding module is included in the model; ✗ indicates that it is excluded.

Model	DG_TA	SA	STAF	SG	DSGCN	PeMS08			PeMS04		
						MAE	RMSE	MAPE (%)	MAE	RMSE	MAPE (%)
w/o DG_TA	✗	✓	✓	✓	✓	15.43	24.79	10.79	19.81	32.25	13.17
w/o SA	✓	✗	✓	✓	✓	15.11	24.35	10.03	19.29	31.94	12.86
w/o STAF	✓	✓	✗	✓	✓	16.43	26.51	10.82	19.88	31.98	13.41
w/o SG	✓	✓	✓	✗	✓	15.05	24.05	10.20	19.16	31.67	12.62
w/o DSGCN	✓	✓	✓	✓	✗	16.83	25.79	11.23	21.34	33.73	14.08
STFDSGCN	✓	✓	✓	✓	✓	14.77	24.05	9.86	19.01	31.27	12.54

**Table 4:** Training and inference time (s/epoch) on the PeMS08 dataset using NVIDIA RTX 4090D GPU.

Model	Training Time (s/epoch)	Inference Time (s)
STSGCN	61.28	12.40
STFGNN	21.93	4.45
STGODE	92.49	8.5
DSTAGNN	134.72	14.94
PDFormer	133.871	8.12
STFDSGCN	27.7	5.6

## Data Availability

Not available.

## References

[1] Jiang R., Yin D., Wang Z., Wang Y., Deng J., Liu H., Cai Z., Deng J., Song X., Shibasaki R. Dl-traff: Survey and benchmark of deep learning models for urban traffic prediction. Proceedings of the 30th ACM International Conference on Information & Knowledge Management.

[2] Wu Z., Pan S., Chen F., Long G., Zhang C., Philip S. (2020). A comprehensive survey on graph neural networks. IEEE Trans. Neural Netw. Learn. Systems.

[3] Chen Y., Shu T., Zhou X., Zheng X., Kawai A., Fueda K., Wang K. (2022). Graph attention network with spatial-temporal clustering for traffic flow forecasting in intelligent transportation system. IEEE Trans. Intell. Transp. Syst..

[4] Williams B., Hoel L. (2003). Modeling and forecasting vehicular traffic flow as a seasonal ARIMA process: Theoretical basis and empirical results. J. Transp. Eng..

[5] Zivot E., Wang J. (2006). Vector autoregressive models for multivariate time series. Modeling Financial Time Series with S-PLUS^®^.

[6] Pascale A., Nicoli M. (2011). Adaptive Bayesian network for traffic flow prediction. Proceedings of the 2011 IEEE Statistical Signal Processing Workshop (SSP).

[7] Wu H., Ho M., Lee T. (2004). Travel-time prediction with support vector regression. IEEE Trans. Intell. Transp. Syst..

[8] Van L., Van H., Chowdhury R., Sadek S. (2012). Short-term traffic and travel time prediction models. Artif. Intell. Appl. Crit. Transp. Issues.

[9] Fu R., Zhang Z., Li L. (2016). Using lstm and gru neural network methods for traffic flow prediction. Proceedings of the 2016 31st Youth Academic Annual Conference of Chinese Association ofAutomation (YAC).

[10] Cho K., Merrienboer B., Gulcehre C., Bahdanau D., Bougares F., Schwenk H., Bengio Y. (2014). Learning phrase representations using rnn encoder-decoder for statistical machine translation. arXiv.

[11] Cui Z., Ke R., Pu Z., Wang Y. (2020). Stacked bidirectional and unidirectional lstm recurrent neural network for forecasting networkwide traffic state with missing values. Transp. Res. Part C Emerg. Technol..

[12] Ma X., Tao Z., Wang Y., Yu H., Wang Y. (2015). Long short-term memory neural network for traffic speed prediction using remote microwave sensor data. Transp. Res. Part C Emerg. Technol..

[13] Chung J., Gulcehre C., Cho K., Bengio Y. (2014). Empirical evaluation of gated recurrent neural networks on sequence modeling. arXiv.

[14] Cui Z., Ke R., Pu Z., Wang Y. (2018). Deep bidirectional and unidirectional LSTM recurrent neural network for network-wide traffic speed prediction. arXiv.

[15] Tian Y., Zhang K., Li J., Lin X., Yang B. (2018). LSTM-based traffic flow prediction with missing data. Neurocomputing.

[16] Xu J., Wang W., Wang H., Guo J. (2020). Multi-model ensemble with rich spatial information for object detection. Pattern Recognit..

[17] Yao H., Tang X., Wei H., Zheng G., Li Z. (2019). Revisiting spatial-temporal similarity: A deep learning framework for traffic prediction. Proc. AAAI Conf. Artif. Intell..

[18] Bai S., Kolter J., Koltun V. (2018). An empirical evaluation of generic convolutional and recurrent networks for sequence modeling. arXiv.

[19] Zhang J., Zheng Y., Qi D. Deep spatio-temporal residual networks for citywide crowd flows prediction. Proceedings of the AAAI Conference on Artificial Intelligence.

[20] Wu Z., Pan S., Long G., Jiang J., Zhang C. (2019). Graph wavenet for deep spatial-temporal graph modeling. arXiv.

[21] Huang R., Huang C., Liu Y., Dai G., Kong W. (2020). LSGCN: Long short-term traffic prediction with graph convolutional networks. IJCAI.

[22] Zhao Y., Lin Y., Wen H., Wei T., Jin X., Wan H. (2022). Spatial-temporal position-aware graph convolution networks for traffic flow forecasting. IEEE Trans. Intell. Transp. Syst..

[23] Cirstea R., Yang B., Guo C., Kieu T., Pan S. (2022). Towards spatio-temporal aware traffic time series forecasting. Proceedings of the 2022 IEEE 38th International Conference on Data Engineering (ICDE).

[24] Liu H., Zhu C., Zhang D., Li Q. (2023). Attention-based spatial-temporal graph convolutional recurrent networks for traffic forecasting. Proceedings of the International Conference on Advanced Data Mining and Applications.

[25] Yu B., Yin H., Zhu Z. (2018). Spatio-temporal graph convolutional networks: A deep learning framework for traffic forecasting. arXiv.

[26] Li Y., Yu R., Shahabi C., Liu Y. (2017). Diffusion convolutional recurrent neural network: Data-driven traffic forecasting. arXiv.

[27] Zheng C., Fan X., Wang C., Qi J. Gman: A graph multi-attention network for traffic prediction. Proceedings of the AAAI Conference on Artificial Intelligence.

[28] Yuan H., Li G. (2021). A survey of traffic prediction: From spatio-temporal data to intelligent transportation. Data Sci. Eng..

[29] Luo X., Zhu C., Zhang D., Li Q. (2023). Dynamic Graph Convolution Network with Spatio-Temporal Attention Fusion for Traffic Flow Prediction. arXiv.

[30] Song C., Lin Y., Guo S., Wan H. (2020). Spatial-temporal synchronous graph convolutional networks: A new framework for spatial-temporal network data forecasting. Proc. AAAI Conf. Artif. Intell..

[31] Li M., Zhu Z. (2021). Spatial-temporal fusion graph neural networks for traffic flow forecasting. Proc. AAAI Conf. Artif. Intell..

[32] Guo S., Lin Y., Feng N., Song C., Wan H. Attention based spatial-temporal graph convolutional networks for traffic flow forecasting. Proceedings of the AAAI Conference on Artificial Intelligence.

[33] Zhang W., Zhu K., Zhang S., Chen Q., Xu J. (2022). Dynamic graph convolutional networks based on spatiotemporal data embedding for traffic flow forecasting. Knowl.-Based Syst..

[34] Kong J., Fan X., Zuo M., Deveci M., Jin X., Zhong K. (2023). ADCT-Net: Adaptive traffic forecasting neural network via dual-graphic cross-fused transformer. Inf. Fusion.

[35] Bui K., Cho J., Yi H. (2021). Spatial-temporal graph neural network for traffic forecasting: An overview and open research issues. Appl. Intell..

[36] Bai L., Yao L., Li C., Wang X., Wang C. Adaptive graph convolutional recurrent network for traffic forecasting. Proceedings of the Advances in Neural Information Processing Systems.

[37] Liu M., Zhu T., Ye J., Meng Q., Sun L., Du B. (2023). Spatio-temporal autoencoder for traffic flow prediction. IEEE Trans. Intell. Transp. Syst..

[38] Xu Y., Liu W., Jiang Z., Xu Z., Mao T., Chen L., Zhou M. (2021). MAF-GNN: Multi-adaptive spatiotemporal-flow graph neural network for traffic speed forecasting. arXiv.

[39] Wang S., Zhang Y., Hu Y., Yin B. (2023). Knowledge fusion enhanced graph neural network for traffic flow prediction. Phys. A Stat. Mech. Its Appl..

[40] Tedjopurnomo D., Bao Z., Zheng B., Choudhury F., Qin A. (2020). A survey on modern deep neural network for traffic prediction: Trends, methods and challenges. IEEE Trans. Knowl. Data Eng..

[41] Liu Y., Shao Z., Hoffmann N. (2021). Global attention mechanism: Retain information to enhance channel-spatial interactions. arXiv.

[42] Jiang W., Luo J. (2022). Graph neural network for traffic forecasting: A survey. Expert Syst. Appl..

[43] Chen M., Han L., Xu Y., Zhu T., Wang J., Sun L. (2024). Temporal-aware structure-semantic-coupled graph network for traffic forecasting. Inf. Fusion.

[44] Kipf T., Welling M. (2017). Semi-supervised classification with graph convolutional networks. arXiv.

[45] Ba J., Kiros J., Hinton G. (2016). Layer normalization. arXiv.

[46] Wang K., An J., Zhou M., Shi Z., Shi X., Kang Q. (2022). Minority-weighted graph neural network for imbalanced node classification in social networks of internet of people. IEEE Internet Things J..

[47] Ding W., Zhang T., Wang J., Zhao Z. (2023). Multi-graph Spatio-temporal Graph Convolutional Network for Traffic Flow Prediction. arXiv.

[48] Fang Z., Long Q., Song G., Xie K. Spatial-temporal graph ode networks for traffic flow forecasting. Proceedings of the 27th ACM SIGKDD Conference on Knowledge Discovery & Data Mining.

[49] Chen Y., Li K., Yeo C., Xie K. (2023). Traffic forecasting with graph spatial–temporal position recurrent network. Neural Netw..

[50] Jiang J., Han C., Zhao W., Wang J. (2023). Pdformer: Propagation delay-aware dynamic long-range transformer for traffic flow prediction. Proc. AAAI Conf. Artif. Intell..

